# Targeting the NF-κB Pathway as a Combination Therapy for Advanced Thyroid Cancer

**DOI:** 10.1371/journal.pone.0134901

**Published:** 2015-08-11

**Authors:** Nikita Pozdeyev, Adam Berlinberg, Qiong Zhou, Kelsey Wuensch, Hiroyuki Shibata, William M. Wood, Bryan R. Haugen

**Affiliations:** 1 Department of Medicine, Division of Endocrinology, Metabolism, and Diabetes, University of Colorado, Anschutz Medical Campus, Aurora, CO, United States of America; 2 Department of Pharmaceutical Sciences, University of Colorado, Anschutz Medical Campus, Aurora, CO, United States of America; 3 Department of Clinical Oncology, Faculty of Medicine, Akita University, Akita, Japan; 4 University of Colorado Cancer Center, Anschutz Medical Campus, Aurora, CO, United States of America; Universite Libre de Bruxelles (ULB), BELGIUM

## Abstract

NF-κB signaling plays an important role in tumor cell proliferation, cell survival, angiogenesis, invasion, metastasis and drug/radiation resistance. Combination therapy involving NF-κB pathway inhibition is an attractive strategy for the treatment of advanced forms of thyroid cancer. This study was designed to test the efficacy of NF-κB pathway inhibition in combination with cytotoxic chemotherapy, using docetaxel and ionizing radiation in *in vitro* models of thyroid cancer. We found that while both docetaxel and ionizing radiation activated NF-κB signaling in thyroid cancer cells, there was no synergistic effect on cell proliferation and/or programmed cell death with either genetic (transduction of a dominant negative mutant form of IκBα) or pharmacologic (proteasome inhibitor bortezomib and IKKβ inhibitor GO-Y030) inhibition of the NF-κB pathway in thyroid cancer cell lines BCPAP, 8505C, THJ16T and SW1736. Docetaxel plus bortezomib synergistically decreased *in vitro* invasion of 8505C cells, but not in the other cell lines. Screening of a panel of clinically relevant targeted therapies for synergy with genetic NF-κB inhibition in a proliferation/cytotoxicity assay identified the histone deacetylase (HDAC) inhibitor suberoylanilide hydroxamic acid (SAHA) as a potential candidate. However, the synergistic effect was confirmed only in the BCPAP cells. These results indicate that NF-κB inhibitors are unlikely to be beneficial as combination therapy with taxane cytotoxic chemotherapy, external radiation therapy or radioiodine therapy. There may be unique circumstances where NF-κB inhibitors may be considered in combination with docetaxel to reduce tumor invasion or in combination with HDAC inhibitors to reduce tumor growth, but this does not appear to be a combination therapy that could be broadly applied to patients with advanced thyroid cancer. Further research may identify which subsets of patients/tumors may respond to this therapeutic approach.

## Introduction

Thyroid cancer is a major public health problem with > 60,000 estimated new cases causing approximately 2000 deaths in the United States in the year 2015 [1]. Anaplastic thyroid cancer (ATC) is the deadliest form of thyroid cancer with a median survival of 5 months and a 20% 1-year survival despite an aggressive management with a combination of surgery, radiation and chemotherapy [2]. Cytotoxic chemotherapy with docetaxel is one of the few treatments that showed encouraging results in ATC, especially when combined with radiation therapy [3].

NF-κB is a family of transcription factors implicated in various aspects of the tumor biology such as cell proliferation, survival, angiogenesis, invasion, metastasis and drug resistance [4]. NF-κB signaling was found to be constitutively activated in hepatocellular carcinoma [5], colorectal cancer [6], breast cancer [7], prostate cancer [8] and other solid and hematologic malignancies [4]. Anti-cancer drugs and ionizing radiation upregulate NF-κB pathway that results in the development of treatment resistance [9,10]. This led to a significant effort to develop NF-κB inhibitors and study of their efficacy for the treatment of cancers both in preclinical models and in clinical trials [4,11].

As has been shown for other tumors, thyroid cancer tissue has increased expression of NF-κB transcription factors that is associated with a larger tumor size, presence of nodal metastases, extrathyroidal extension and *BRAF V600E* mutation [12–15]. The role of NF-κB signaling in thyroid cancer cell growth, migration, invasion and angiogenesis has been shown [16–18]. A synergistic effect of docetaxel and the NF-κB pathway inhibitor curcumin on the proliferation and apoptosis of the ATC cell line 8505C has been reported [19]. The combination of the I^131^ and NF-κB inhibition with either an IκB kinase (IKK) inhibitor Bay 11–7082 or p65 siRNA resulted in a greater decrease of thyroid cancer growth *in vitro* [20] and in a flank xenograft model in mice [21] than either therapy alone. Encouraged by these findings we designed this study aimed to systematically investigate the benefits of the combination therapy with either docetaxel or ionizing radiation and NF-κB pathway inhibition using *in vitro* models of thyroid cancer.

## Methods

### Cell lines and genetic constructs

**Fig 1:**
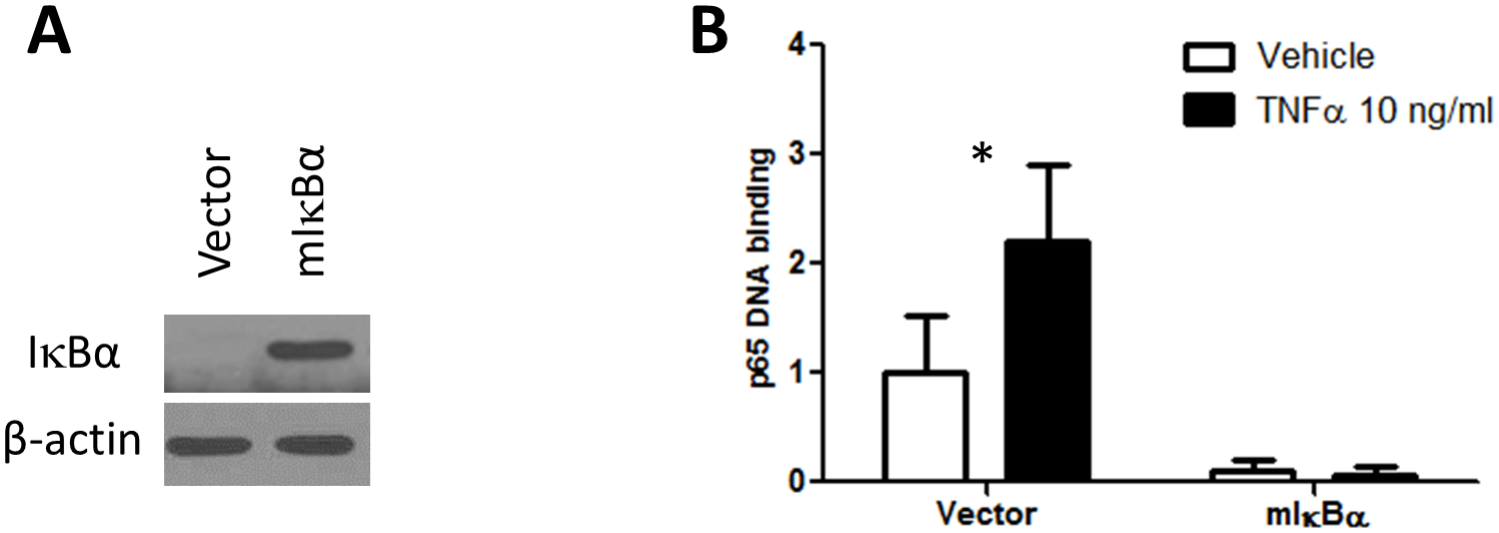
mIκBα transduction of BCPAP cells causes IκBα accumulation (A) and inhibition of both baseline and TNFα-stimulated p65 DNA binding (B). Data are normalized to the vehicle treated vector group. *—p<0.01 for the effect of TNFα in the vector group (two-way ANOVA followed by Bonferroni posttest).

This study was performed using a papillary thyroid cancer (PTC) cell line BCPAP and three ATC cell lines 8505C, THJ16T and SW1736 that have been validated by short tandem repeat profiling [22,23]. Cell lines were engineered to express the dominant negative mutant form of IκBα (mIκBα) that is resistant to proteasomal degradation [24]. The accumulation of vastly excess cytoplasmic mIκBα prevents nuclear translocation of NF-κB transcription factors shutting down canonical NF-κB signaling. Generation of pQCXIP-mIκBα construct, transduction and antibiotic selection are described in detail elsewhere [17]. The phenotype of each cell line was tested by measuring the accumulation of IκBα protein by western blot and functionally by estimating NF-κB transcription factor p65 binding to κB DNA consensus sites (TransAM NFκB p65 ELISA kit, Active Motif). Tumor necrosis factor α (TNFα) was used as a positive control for NF-κB signaling activation. We demonstrated that mIκBα transduction leads to IκBα accumulation and inhibition of both baseline and TNFα-stimulated p65 DNA binding in BCPAP cells (Fig 1) and other thyroid cancer cell lines (data not shown).

**Fig 2:**
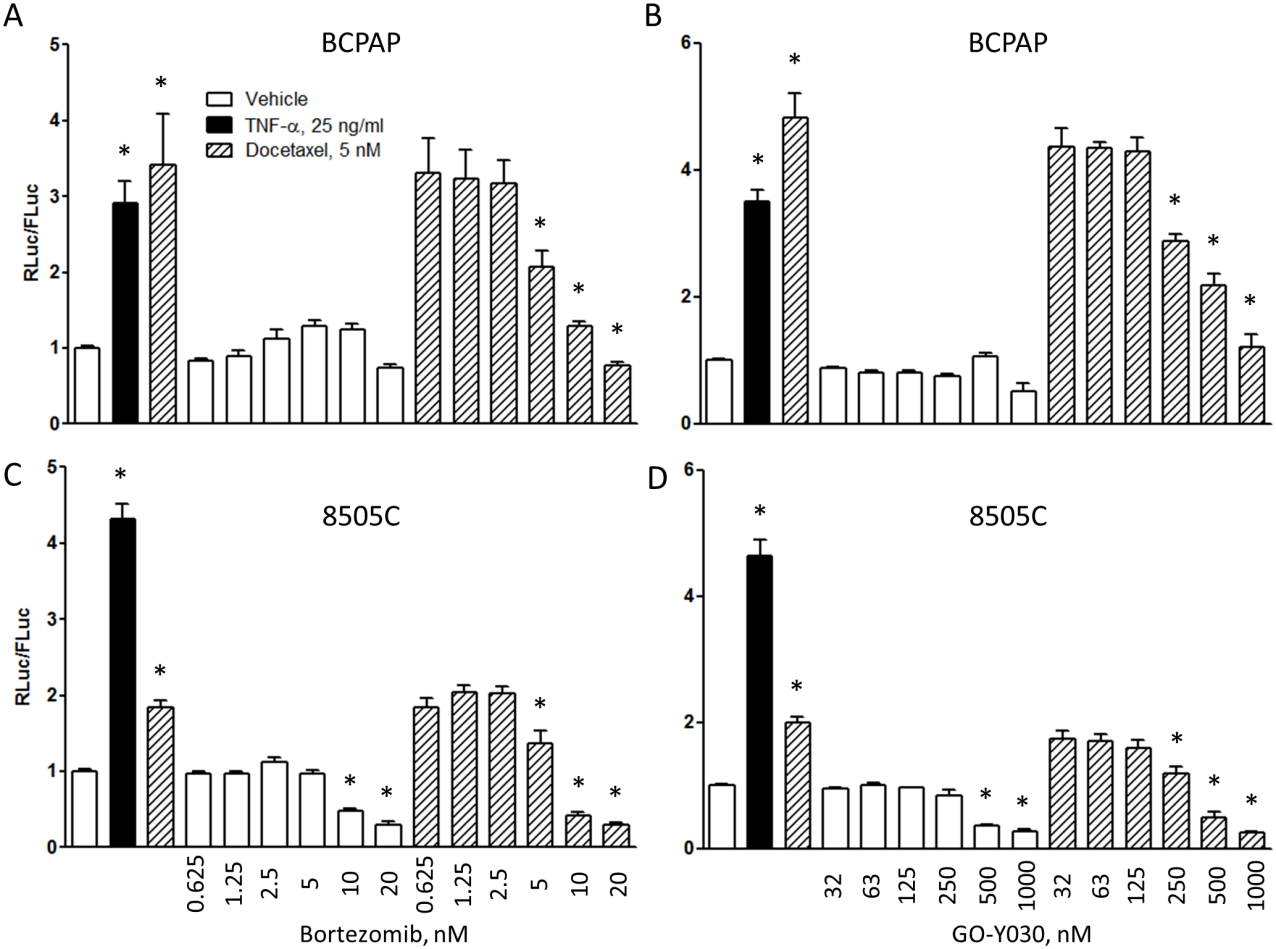
Docetaxel activates NF-κB signaling in thyroid cancer cell lines BCPAP (A,B) and 8505C (C,D). Proteasome inhibitor bortezomib and IKK complex inhibitor GO-Y030 counteract NF-κB activation by docetaxel. Cells were incubated with drugs for 48 h prior to reporter luciferase activity measurements. The differences between groups are highly significant (p<0.0001, one-way ANOVA). Asterisks indicate significant change (p<0.05, Newman-Keuls posttest) when compared to the corresponding control group (TNFα, docetaxel alone and bortezomib alone were compared to vehicle; docetaxel and bortezomib/GO-Y030 combinations were compared to the docetaxel only group).

To conveniently monitor NF-κB pathway activity we constructed a lentiviral vector containing a *Renilla* luciferase reporter driven by a minimal *c-fos* promoter under the control of four upstream NF-κB response elements (κB). This vector was transduced into BCPAP and 8505C thyroid cancer cells already expressing *Firefly* luciferase driven by a constitutive CMV promoter as a non-NF-κB-regulated control. After transduction and antibiotic selection with puromycin, cells were assessed for expression of both *Firefly* and *Renilla* luciferase activity (Fig 2, Dual-Luciferase Reporter Assay System, Promega).

### Drugs and radiation treatments

Most pharmacologic agents were obtained in a powder form from LC Labs and Sellekchem and reconstituted in DMSO. The final concentration of DMSO in the culture media did not exceed 0.05%. The proteasomal inhibitor bortezomib, an FDA approved drug that blocks NF-κB pathway by inhibiting IκBα proteolysis [25], was used to study drug-drug synergy. An experimental IKKβ inhibitor GO-Y030 [26] served as an alternative pharmacologic NF-κB signaling inhibitor and was obtained from Dr. Shibata’s laboratory (Akita, Japan).

X-ray ionizing radiation was delivered at a dose of 2 and 5 Gy by an RS2000 X-ray irradiator (Rad Source Technologies).

### Cell cytotoxicity/proliferation/viability assays

The effects of drugs, ionizing radiation and genetic modification on tumor cells survival and proliferation were measured by 72 h sulforhodamine B assay (SRB) [27] as well as by direct counting of the number of viable cells using a Vi-CELL XR Cell Viability Analyzer (Beckman Coulter).

### *In vitro* invasion assay

Thyroid cancer cells were seeded at a density of 5x10^6^ cells per plate, and grown in RPMI-1640 media supplemented with 5% FBS for 6 hours. Media was then aspirated and replaced with the media supplemented with 0.1% FBS, and cells were incubated for 18 hours. Cells were harvested and 1x10^6^ cells were seeded in the upper chambers of Matrigel-coated transwell chambers (24-well, 8 μm pore size; BD Biosciences) in RPMI-1640 with 0.1% FBS. Assays were carried out in duplicate. Cells invaded into the lower chamber containing media with 10% FBS. After 18 hours, non-invading cells in the top chamber were discarded, and invading cells were fixed with methanol, stained with 3 μg/mL 4’,6-diamidino-2-phenylindole (DAPI; Invitrogen) and counted in five microscopic fields under 10x magnification using Metamorph software (Molecular Devices).

### Screening of clinically relevant targeted drugs for the synergy with NF-κB pathway inhibition

Proliferation of cells expressing mIκBα (as a genetic model of NF-κB pathway inhibition, shown to produce a highly effective phenotype (Fig 1)) was assessed by SRB assays. Three thyroid cancer cell lines were exposed to a number of cytotoxic and targeted therapies. Of the 15 targeted therapies used for the screening, 12 were kinase inhibitors, DAPT is a γ-secretase inhibitor, decitabine is a DNA methyltrasferase inhibitor, and suberoylanilide hydroxamic acid (SAHA, vorinostat) inhibits histone deacetylases. Eight point dose response curves were obtained and half maximal inhibitory drug concentration (IC_50_) was calculated by fitting the experimental data using 4-parameter logistic nonlinear regression with the aid of a custom made R script. Hits were identified by comparing IC_50_ for control vector and mIκBα transduced cells and were further evaluated using SRB assay and viable cells counting.

### Caspase-3/7 assay

Cells were seeded in 96-well plates in duplicate at a density of 7.5x10^4^ cells/well in RPMI-1640 media supplemented with 5% fetal bovine serum (FBS). Following overnight incubation drugs were added either alone or in combination for 24 hours and FBS in the media was changed to 0.1%. Caspase-3/7 activity was assessed using a luminescence-based caspase assay according to the manufacturer instructions (Caspase GloTM 3/7 Assay, GloMax Multimode Reader, Promega).

### Statistical and synergy calculations

Statistical calculations were done using GraphPad Prism. The specific analysis method used and the calculated p-values are outlined in a corresponding figure legend. Data are expressed as the mean ± standard deviation.

Bliss independence index (BI) and combination index (CI) were calculated as a measure of the synergistic effect. A BI and CI <0.8 were accepted as an evidence of synergy. BI was calculated using the following formula: BI = ((F_a_+F_b_)-(F_a_*F_b_))/F_ab_, where F_a_ is the effect of a drug or radiation treatment; F_b_ is the effect of a pharmacologic NF-κB inhibitor or mIκBα; (F_a_+F_b_)-(F_a_*F_b_) is the predicted sum of the effects of two treatments; F_ab_ is the actual effect of a combination therapy found experimentally.

Drug/drug synergy was also evaluated by the Chou combination index (CI) [28] using Compusyn software (http://www.combosyn.com). For these calculations we constructed 8-point dose response curves for single drug and drugs combination. CI for IC_50_ is reported.

## Results

### Docetaxel and ionizing radiation activate NF-κB pathway in thyroid cancer cells

Using BCPAP and 8505C cells engineered to express *Renilla* luciferase NF-κB activity reporter as a readout of NF-κB activity, both positive control 25 ng/ml TNFα and 5 nM docetaxel for 48 h increased NF-κB reporter activity (Fig 2). The effect of docetaxel was greater in BCPAP cells (Fig 2A and 2B). Pharmacologic NF-κB pathway inhibitors were effective in abrogating the docetaxel-induced increase in reporter activity at concentrations of ≥ 5 nM and ≥ 250 nM for bortezomib (Fig 2A and 2C) and GO-Y030 (Fig 2B and 2D), respectively.

**Fig 3:**
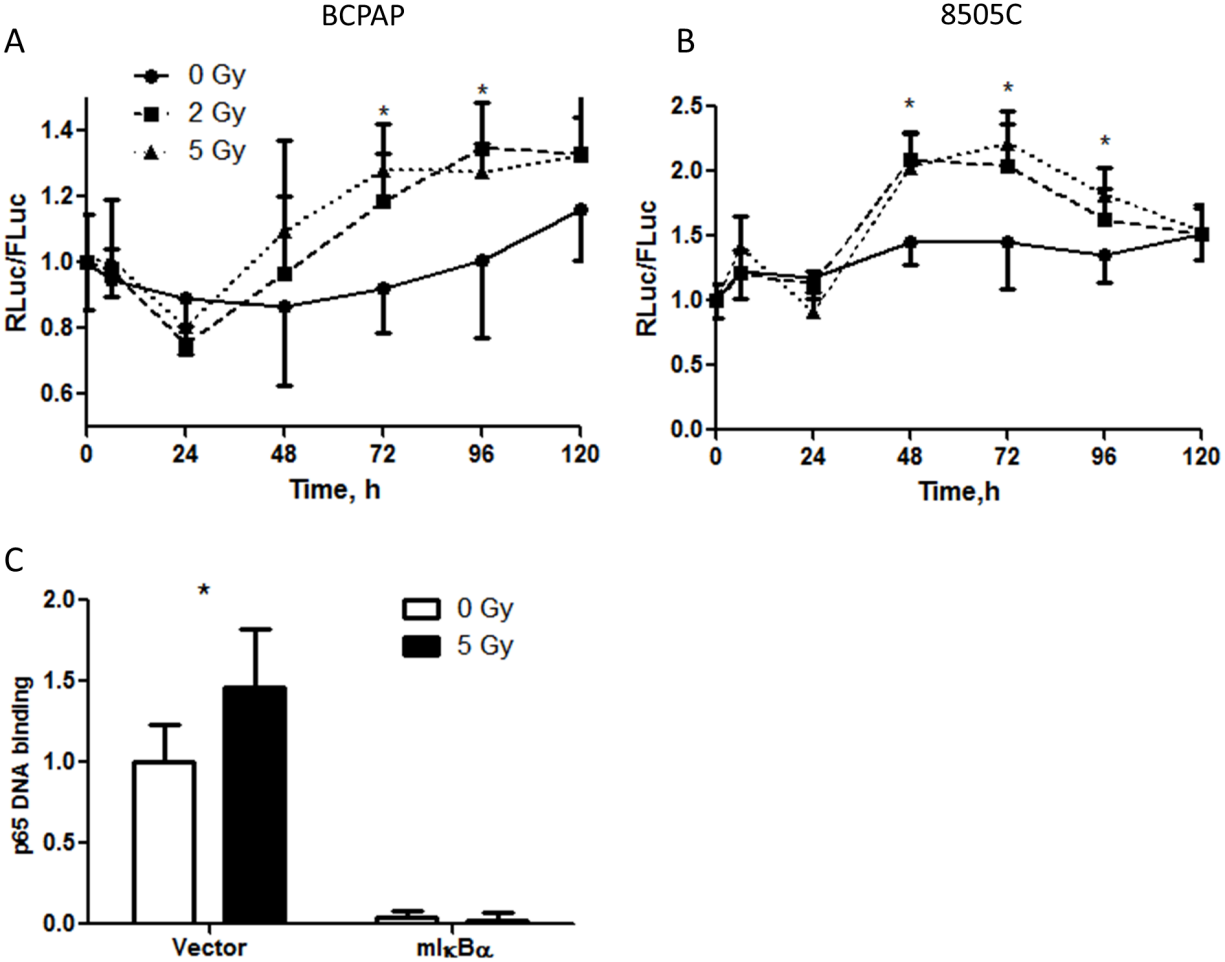
The ionizing radiation activates NF-κB signaling in BCPAP (A,C) and 8505C (B) thyroid cancer cells as measured by dual luciferase reporter assay (A, B) and p65 DNA binding ELISA (C). p65 DNA binding experiment was performed 72 h after radiation exposure. Data are normalized to non-irradiated group (at a time 0 h for panels A and B). *—p<0.05, (two-way ANOVA, Bonferroni posttest).

A time course of the effect of ionizing radiation on NF-κB activity luciferase reporter in BCPAP and 8505C cells is shown in Fig 3A and 3B. Similar to docetaxel, NF-κB pathway was activated after ≥ 48 h exposure to ionizing radiation. This finding was confirmed using a functional assay which measured p65 binding to DNA segments containing NF-κB sequences in BCPAP cells (Fig 3C). In mIκBα overexpressing cells, both baseline and radiation-induced NF-κB signaling were blocked.

### Lack of a synergistic effect of docetaxel or ionizing radiation and pharmacologic or genetic NF-κB pathway inhibition on thyroid cancer cells proliferation/death

The next series of experiments tested the hypothesis that the effect of clinically relevant treatments such as docetaxel and ionizing radiation known to activate NF-κB signaling is potentiated by pharmacologic (bortezomib and GO-Y030) or genetic (mIκBα) NF-κB signaling inhibition. No synergy was found irrespective of the synergy calculation method, cytotoxicity/cell viability assay or thyroid cancer cell line used (Table 1). Similarly, no synergy was found when ionizing radiation at 2 and 5 Gy doses was combined with NF-κB blockade by mIκBα for 48 or 72 h in BCPAP and SW1736 cells (Table 2).

**Table 1:** Lack of the synergistic effect of docetaxel and pharmacologic or genetic NF-κB pathway inhibition on thyroid cancer cell death/proliferation.

Cell line	Method	mIkBα	Bortezomib		GO-Y030	
		BI	BI	CI	BI	CI
BCPAP	SRB		0.94	1.35	0.84	1.45
BCPAP	Vi-CELL	1.11			0.92	
8505C	SRB		1.03	1.31		
THJ16T	SRB		1.07	1.10	0.89	1.16
SW1736	SRB		1.07	1.10	0.89	1.16

BI—Bliss independence index, CI—combination index, SRB—sulforhodamine B cytotoxicity/proliferation assay; ViCell—cell viability assay.

**Table 2:** Lack of the synergistic effect of ionizing radiation and NF-κB pathway inhibition with mIκBα on thyroid cancer cell death/proliferation.

Cell line	Method	2 Gy		5 Gy
		48 h	72 h	72 h
BCPAP	Vi-CELL	1.11	0.91	1.19
SW1736	Vi-CELL	0.87	0.89	1.07

Cells were exposed to 2 or 5 Gy of X-rays and the number of viable cells was estimated 48 or 72 h after the exposure. Vi-CELL—cell viability assay.

### Docetaxel does not activate caspase-3/7 in thyroid cancer cells

**Fig 4:**
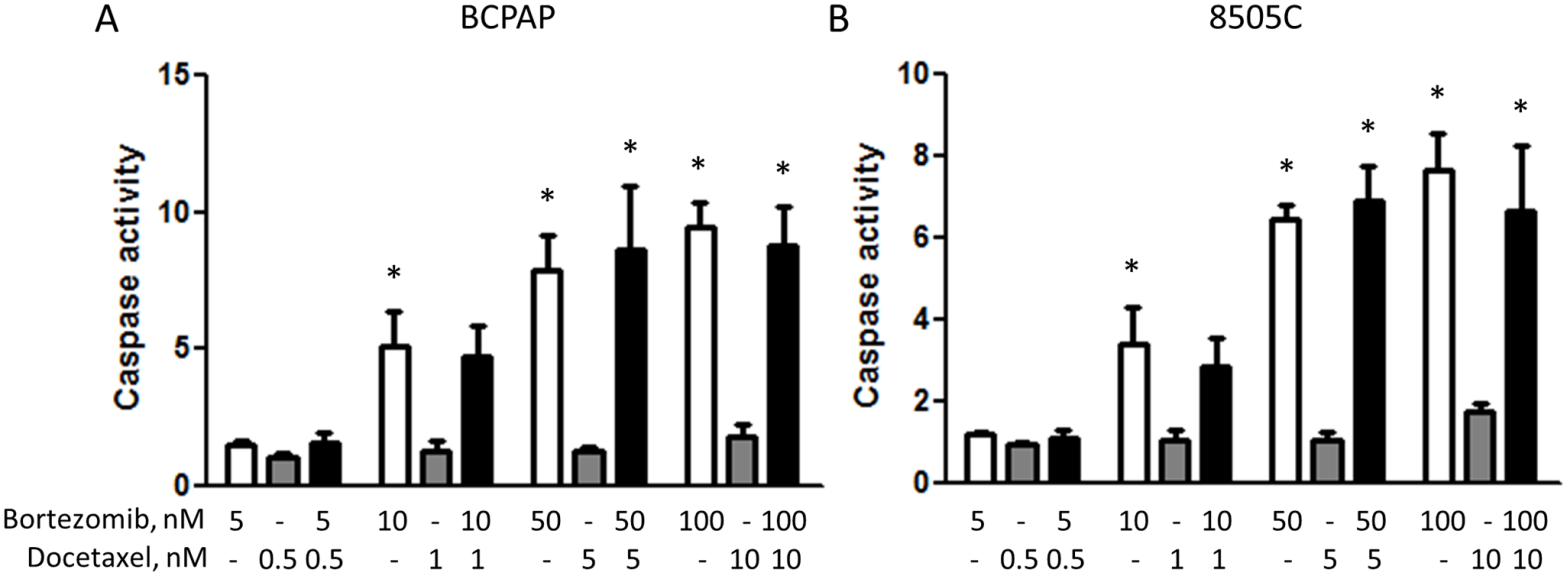
The caspase-3/7 activity is increased in BCPAP (A) and 8505C (B) thyroid cancer cells by bortezomib and a combination of bortezomib and docetaxel but not when treated with docetaxel alone. Asterisks point to a statistical significance when compared to a lowest dose group (p<0.05, one-way ANOVA, Newman-Keuls posttest).

NF-κB signaling inhibition sensitizes cells to apoptosis induced by cytokines such as TNFα [24]. It has been reported that docetaxel also induces apoptosis in thyroid cancer cells [29] but it failed to show synergy with NF-κB pathway inhibition in our experiments. Taking this discrepancy into consideration we decided to assess whether docetaxel induces apoptosis in our models. Contrary to published data, docetaxel, while being highly effective in SRB assay (IC_50_ in picomolar range, data not shown), failed to induce caspase-3/7 activity in BCPAP and 8505C cells (Fig 4). Bortezomib, used as a positive control, dose dependently activated caspase-3/7 in these cell lines.

### The effect of docetaxel and bortezomib on thyroid cancer cell invasion *in vitro*

**Fig 5:**
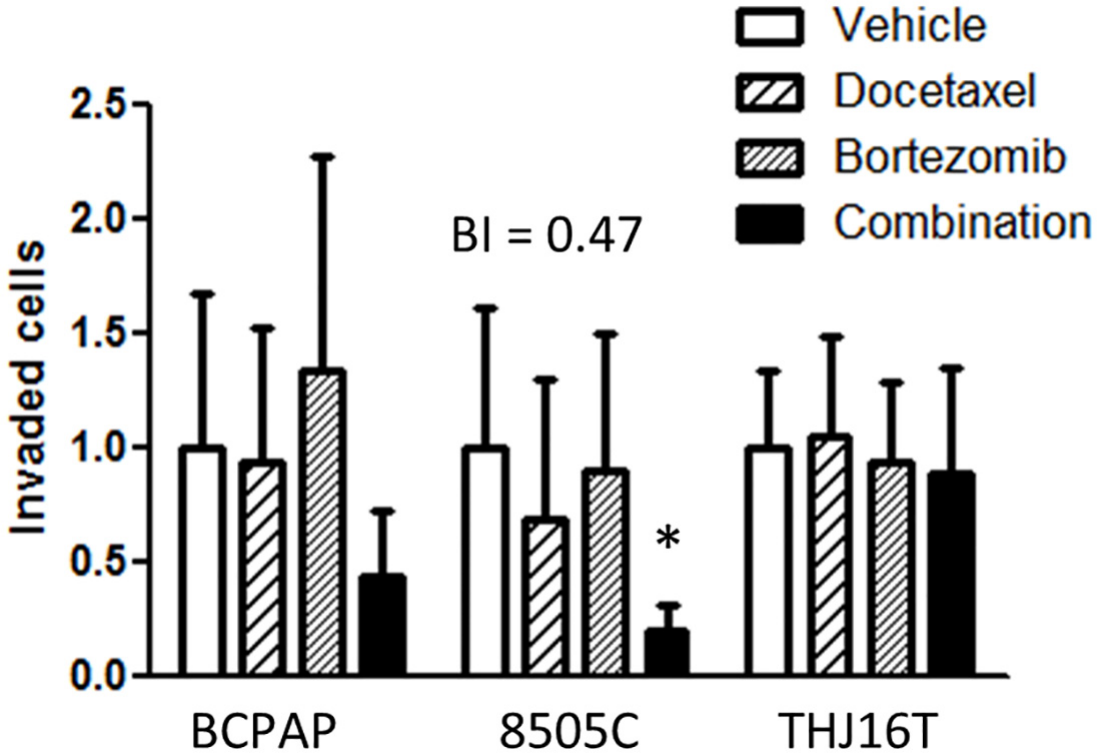
The docetaxel and bortezomib synergistically decrease thyroid cancer cell line 8505C invasion *in vitro*. Asterisks indicate a significant effect of the drug combination (p<0.05, repeated measurements one-way ANOVA, Newman-Keuls posttest). The effect of the drugs on the invasion of BCPAP and THJ16T cells was not significant. BI—Bliss independence index.

The effect of a docetaxel/bortezomib combination on a more complex aspect of the tumor biology, matrix invasion, was tested (Fig 5). The drug combination synergistically inhibited 8505C thyroid cancer cell invasion *in vitro* (BI of 0.47 suggests a moderate degree of synergy) but had no effect on invasion of THJ16T cells. A trend towards decreased invasion in BCPAP cells after treatment with docetaxel/bortezomib combination was not significant.

### The screening for drugs which cytotoxic/anti-proliferative effect on thyroid cancer cells is potentiated by NF-κB pathway inhibition with mIκBα

We have extended our efforts to find drug/NF-κB inhibition synergy by performing a screening using a series of clinically relevant targeted drugs. mIκBα was selected as a “clean” method of NF-κB pathway inhibition that is less likely to produce off-target effects as compared to pharmacologic NF-κB inhibitors. Three cell lines were engineered to express mIκBα and the phenotype was confirmed as described in the methods section and shown on the Fig 1. The SRB assay IC_50_ values are summarized in the Table 3. Five drugs (cabozantinib, dabrafenib, DAPT, selumetinib and vandetanib) were ineffective at maximally tested concentrations and IC_50_ could not be calculated. Everolimus was the most potent drug. Among the three cell lines, BCPAP was more sensitive and 8505C more resistant overall and this correlated with the rate of proliferation (data now shown). SAHA was the only compound that consistently showed synergy in combination with mIκBα and was further studied.

**Table 3:** IC_50_ for 15 targeted therapies in vector and mIκBα transduced thyroid cancer cell lines

Drug	IC_50_ (nM)					
	BCPAP		8505C		THJ16T	
	Vector	mIκBα	Vector	mIκBα	Vector	mIκBα
Axitinib	3155	2358	4971	3545	709	354
Bosutinib	359	315	1474	1343	757	679
Cabozantinib	>1000	>1000	>1000	>1000	>1000	>1000
Dabrafenib	>5000	>5000	>5000	>5000	>5000	>5000
DAPT	>20000	>20000	>20000	>20000	>20000	>20000
Dasatinib	12	49	>1000	980	112	112
Decitabine	8691	9042	5479	1578	692	1009
Everolimus	0.38	0.37	0.58	0.31	0.51	0.48
Pazopanib	3257	4071	2816	4024	2781	2138
Ponatinib	122	157	367	398	263	245
Regorafenib	2509	2613	3640	4328	2728	2896
SAHA	146	47	309	189	344	168
Saracatinib	1316	635	>1000	>1000	>1000	>1000
Selumetinib	>1000	>1000	>1000	>1000	>1000	>1000
Vandetanib	>1000	>1000	>1000	>1000	>1000	>1000

**Fig 6:**
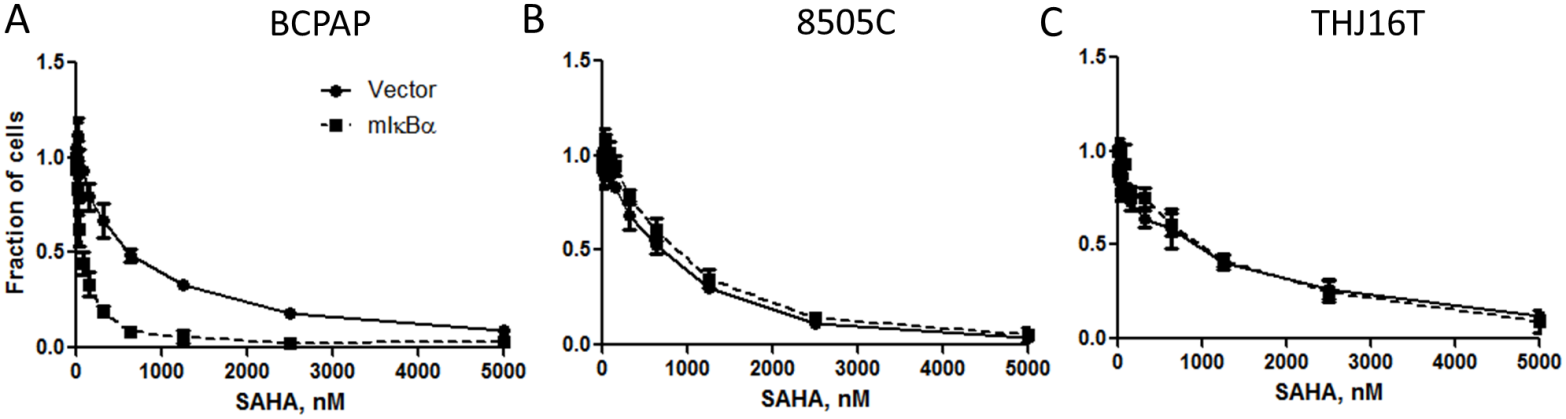
NF-κB signaling inhibition with mIκBα potentiates cytotoxic/anti-proliferative effect of SAHA in BCPAP (A) but not 8505C (B) and THJ16T (C) thyroid cancer cells. The differential response to SAHA in BCPAP cells was significant at p <0.0001 (two-way ANOVA).

The potentiation of SAHA cytotoxic/anti-proliferative effect by mIκBα was confirmed in a 12-concentration SRB assay in BCPAP cells but not in 8505C or THJ16T cells (Fig 6). The synergy was also demonstrated by direct counting of viable cells after 3 days of exposure to 500 nM of SAHA (BI = 0.69 and 1.18 for BCPAP and 8505C cells, respectively).

## Discussion

In this study, we sought to determine if inhibition of the NF-κB pathway could synergistically inhibit thyroid cancer cell growth using *in vitro* thyroid cancer models. Docetaxel and radiation activate the NF-κB pathway in thyroid cancer cells confirming earlier observations [19,20,29]. However, despite measuring cytotoxicity/proliferation, viability as well as invasion of thyroid cancer cells, using both pharmacologic and genetic NF-κB pathway inhibition and employing two algorithms for the computation of the combination effect, the evidence for synergistic activity was limited. Specifically, it was found that docetaxel and bortezomib synergistically decreased *in vitro* invasion in 8505C cells only. Out of the 16 drugs (including docetaxel and 15 other drugs in the screening) only SAHA synergistically reduced cell growth when the NF-κB pathway was blocked, and this effect was limited to one cell line (BCPAP).

The apparent discrepancy between our current findings and previously published studies of docetaxel [19] and I^131^ [20,21] is likely explained by the differences in the methodology of establishing the synergistic effect. True synergy occurs when the result of the combination treatment *exceeds* that of the *sum* of the effects of each individual treatment and this has to be proven quantitatively. To our knowledge none of the previously published studies addressed the question of synergy with NF-κB signaling inhibition using the same quantitative approach as in this study.

Increased NF-κB signaling in cancer cells is known to block apoptosis [30]. NF-κB pathway inhibition has been shown to sensitize cells to the pro-apoptotic effect of TNFα [24]. We speculate that the high potency of SAHA in synergy assay is a result of the pro-apoptotic activity of histone deacetylase inhibitors [31]. The lack of apoptosis induction by docetaxel and the poor performance of this agent in this synergy study provide further evidence supporting this hypothesis. However, a detailed experimental investigation of the role of apoptosis in the drug/NF-κB pathway inhibition synergy is beyond the scope of this focused project. The SAHA effect potentiation by NF-κB pathway inhibition may be of clinical interest. However, the enthusiasm is diminished by the fact that this effect was observed in only one out of three *in vitro* thyroid cancer models (and the mechanisms responsible for this selectivity are unclear). In addition, SAHA monotherapy was not effective in a phase II trial of patients with metastatic radioiodine-refractory thyroid carcinoma [32].

This study investigated several aspects of tumor biology such as cell survival, proliferation and invasion that can be conveniently measured *in vitro*. It remains possible that the anti-tumor benefit of NF-κB pathway inhibition may be mediated by more complex processes that are difficult to study in cell lines, such as angiogenesis, immune response and others. Also, thyroid cancer cells cultured *in vitro* are dedifferentiated [33] and may lack phenotypic features that are important for the synergistic action of NF-κB signaling inhibitors.

We did demonstrate synergistic effect of docetaxel and bortezomib on thyroid cancer cell invasion, but this was restricted to only one cell line (8505C), which again makes any consideration of this combination therapy in advanced thyroid cancer less appealing.

In summary, it was found that combination therapy involving NF-κB pathway inhibitors is of limited benefit in thyroid cancer. There may be as yet undiscovered circumstances where NF-κB inhibitors can be considered in combination with docetaxel to reduce tumor invasion or in combination with HDAC inhibitors to reduce tumor growth, but this does not appear to be a combination therapy that could be broadly applied to patients with advanced thyroid cancer. Further research may identify which subsets of patients/tumors may respond to this therapeutic approach.
